# Effect of dietary mannan oligosaccharides and fructo-oligosaccharides on physico-chemical indices, antioxidant and oxidative stability of broiler chicken meat

**DOI:** 10.1038/s41598-021-99620-2

**Published:** 2021-10-18

**Authors:** Avishek Biswas, Namit Mohan, Kapil Dev, Nasir Akbar Mir, Ashok Kumar Tiwari

**Affiliations:** Avian Nutrition and Feed Technology Division, ICAR-Central Avian Research Institute, Izatnagar, Bareilly, Uttar Pradesh 243122 India

**Keywords:** Biochemistry, Biotechnology, Microbiology, Zoology

## Abstract

The objective of this present study was to investigate the potentiality of prebiotics (mannan oligosaccharides-MOS and fructo-oligosaccharides-FOS) in replacement of antibiotic growth promoter and their relationship with physico-chemical indices, antioxidant and oxidative stability and carcass traits of broiler chickens meat. Accordingly, 240 day-old broiler chicks of uniform body weight divided in 6 treatment groups with 5 replicate each (5 × 6 = 30) having 8 birds in each replicate. Six corn based dietary treatments were formulated viz. T_1_ (control diet), T_2_ (T_1_ + Bacitracin methylene di-salicylate @ 0.002%), T_3_ (T_1_ + 0.1% MOS), T_4_ (T_1_ + 0.2% MOS), T_5_ (T_1_ + 0.1% FOS), and T_6_ (T_1_ + 0.2% FOS). Significant (*p* < 0.05) increase in cut up part yields (%) and reduction in cholesterol and fat content in T_4_ (0.2% MOS) group. The water holding capacity (WHC) and extract release volume (ERV) were increase (*p* < 0.05) in 0.1 or 0.2% MOS supplemented group. DPPH (1, 1-diphenyl-2-picrylhydrazy) was higher (*p* < 0.05) and lipid oxidation (free fatty acid and thio-barbituric acid reactive substances) was lower (*p* < 0.05) in T_4_ group. The standard plate count (SPC), staphylococcus and coliform counts were decreased (*p* < 0.05) in T_3_ or T_4_ group. Thus, it can be concluded that mannan oligosaccharides (MOS) may be incorporated at 0.2% level in diet for improved physico-chemical indices, antioxidant and oxidative stability and carcass characteristics of broiler chickens meat and it may be suitable replacer of antibiotic growth promoter.

## Introduction

It is estimated that 70–75% of poultry production costs are incurred by feed cost, which is constantly increasing. The cost of poultry food can be drastically reduced with precise nutrition supply to the feed. Since 1940, antibiotic growth promoters (AGPs) have been widely used to build the immune-competence of birds against different infectious diseases. In intensive poultry production systems, using AGPs has demonstrated a positive impact on chicken growth and feed efficiency by improving gut health and lowering the incidence of sub-clinical infections. Using antibiotics for extensive terms may lead to the development of bacteria resistant to drugs, which can be transferred to humans^1^. So, the World Health Organization (WHO) and the Economic and Social Committee of the European Union (ESCEU) established that the use of antimicrobials in food animals is a public health concern^2^. In addition to preventing intestinal thickening antibiotics also enhance nutrient absorption by reducing the competition between the microbes and the host^3^. There is a high level of pessimism regarding the use of AGPs in the poultry feed industry at sub-therapeutic doses because AGPs have been strongly linked to antibiotic-resistant pathogens, which may pose a threat to human health^4^. The microbial meat quality is another important issue that has to be considered very seriously as the enteropathogens like *Escherichia coli* and *Staphylococci* has the public health hazard and food borne intoxication^5^. The World Health Organization (WHO) reported that the world population is now 7.8 billion, and 56 million people die every year; where 7.69% of people experience foodborne diseases, and 7.5% of annual deaths, i.e., 56 million deaths, were caused by foodborne illness in the world^6^.

Natural feed additives, such as prebiotic have a potential to cut foodborne pathogen load in poultry and the next contamination of poultry products^7^. Prebiotics are non-digestive carbohydrates that can help to stimulate the growth and activity of beneficial bacteria in the gut and are defined as ‘a selectively fermented ingredient that allows specific changes, both in the composition and/or activity in the gastrointestinal microflora that confers benefits upon host well-being and health’^8^.

Prebiotics have the ability to increase the levels of health-promoting bacteria in the intestinal tract and when the prebiotic reaches in the colon, certain members of the indigenous microflora ferment it selectively. The usual target for prebiotics is the two lactic acid bacterial genera, *Bifidobacterium* and *Lactobacillus*. The enhancement of the growth of these bacterial species also results in the production of bacteriocins, which help prevent the growth of pathogenic bacteria^2,9^. One of the major causes to deterioration of meat quality and reduction of the shelf life for meat and meat products is the susceptibility of the lipid macronutrients to various medications. Prebiotics can alter lipid metabolism and enhance the polyunsaturated fatty acids (PUFAs) ratio in chicken meat with benefits to human health. Oxidations of lipids of meat have a negative effect on the consumers and will ultimately lead to economic losses^10^. The most common prebiotics are oligosaccharides, which are naturally occurring carbohydrates found in foods such as fruit, vegetables, including leeks and artichokes and cereals etc. Whereas, synthetically derived ingredients are as yet few, and they are generally termed as galacto oligosaccharides (GOS). In the present study, two natural derived prebiotics MOS and FOS have been used for observing the physico-chemical properties of broiler meat.

A number of studies have supported the beneficial effects of using prebiotics in improving the animal health and production^11,12^. However, the evaluation of beneficial effects of these feed additives must not be limited to growth performance only, but should also include the quality and safety aspects of meat. A number of research results are available on this subject, but there is no consensus between these results. Some researchers deliberate that feeding of prebiotic have been useful to improve the meat and carcass quality^13^, since, others have refuted such results^10^.

Thus, the objective of this experiment was to investigate the effect prebiotics (mannan oligosaccharides-MOS and fructo-oligosaccharides-FOS) in replacement of antibiotic growth promoter and their relationship with physico-chemical indices, antioxidant and oxidative stability and carcass characteristics of broiler chickens meat.

## Results

### Carcass traits

The effect of prebiotics on carcass characteristics and cut-up parts are presented in Tables 1 and 2. Significant (*p* > 0.05) difference was not found in dressed and eviscerated yields, while, substantially (*p* < 0.05) better thigh, breast, back, and drumstick weights (% of live weight) were recorded in T_4_ (0.2% MOS) group followed by statistically comparable T_3_ (0.1% MOS) as compared to control, antibiotic, and other prebiotic supplemented groups (Table 2). Similarly, considerable (*p* > 0.05) differences were not recorded in neck, wing, and organ (heart, liver and gizzard) weights among the dietary supplemented groups.

**Table 1:** Effects of dietary inclusion of prebiotics on carcass characteristics and organ weight (% of live weight) in broiler chickens.

Group parameters	T1	T2	T3	T4	T5	T6	SEM	*p* Value
Dressing yield	71.34	70.23	72.34	72.24	71.32	71.16	3.25	NS
Eviscerated yield	65.45	65.74	67.82	65.66	65.68	65.56	2.32	NS
Heart	0.50	0.49	0.54	0.55	0.53	0.51	0.001	NS
Liver	2.25	2.17	2.32	2.26	2.23	2.21	0.002	NS
Gizzard	2.02	1.95	2.08	2.01	1.95	1.98	0.002	NS

T_1_ (no MOS/FOS/BMD), T_2_ (0.002% BMD), T_3_ (T1 + 0.1% MOS), T_4_ (T1 + 0.2% MOS), T_5_ (T1 + 0.1% FOS), T_6_ (T1 + 0.2% FOS).

SEM = Standard error of mean; NS = Non-significant; n = 15.

Mean values bearing the same superscript in a row did not differ significantly (*p* < 0.05).

**Table 2:** Effects of dietary inclusion of prebiotics on cut up parts (% of live weight) in broiler chickens.

Group parameters	T1	T2	T3	T4	T5	T6	SEM	*p* Value
Thigh	9.82^b^	9.67^b^	10.04^a^	10.45^a^	9.91^b^	9.99^b^	0.37	0.002
Breast	16.54^b^	16.30^b^	17.45^ab^	18.50^a^	17.10^ab^	16.95^b^	0.56	0.011
Back	17.44^b^	17.38^b^	18.20^a^	18.24^a^	18.13^a^	18.05^a^	0.42	0.021
Wings	7.94	7.89	8.07	8.04	8.03	7.99	0.95	0.072
Neck	4.62	4.55	4.70	4.64	4.59	4.50	0.24	0.055
Drumstick	10.10^ab^	09.94^b^	10.84^a^	10.60^a^	10.34^ab^	10.21^ab^	0.48	0.008

T1 (no MOS/FOS/BMD), T2 (0.002% BMD), T3 (T1 + 0.1% MOS), T4 (T1 + 0.2% MOS), T5 (T1 + 0.1% FOS), T6 (T1 + 0.2% FOS).

SEM = Standard error of mean; NS = Non-significant; n = 15.

Mean values bearing the same superscript in a row did not differ significantly (*p* < 0.05).

### Physico-chemical indices

The results of physico-chemical parameters as affected by feeding prebiotics to broiler chicken shown Table 3 indicated cholesterol and fat content of meat been significantly (*p* < 0.05) in birds fed 0.2% MOS (T4 group) which changed into statistically alike to MOS and FOS supplemented birds. The cholesterol and fat content of meat was higher in birds fed control diet (T_1_ group) or BMD supplemented diet (T_2_ group) which was statistically similar to T_3_, T_5_, and T_6_ groups. The pH and drip loss (%) of chicken meat were not significantly (*p* > 0.05) influenced by dietary treatments. Significantly higher (*p* < 0.05) WHC and ERV of chicken meat was observed in birds fed 0.2% MOS (T_4_ group) or 0.1% MOS (T_3_ group) which were statistically similar to WHC and ERV of meat from FOS fed birds (T_5_ and T_6_ groups). The meat from birds fed control diet (T_1_ group) or BMD supplemented diet (T_2_ diet) revealed lower WHC and ERV values which did not differ significantly from FOS supplemented birds.

**Table 3:** Effects of dietary inclusion of prebiotics on physio- biochemical characteristics of meat in broiler chickens.

Group parameters	T1	T2	T3	T4	T5	T6	SEM	*p* Value
pH	5.55	5.56	5.59	5.57	5.60	5.58	0.07	0.078
WHC^#^ (%)	43.14^b^	43.32^b^	46.53^b^	47.17^a^	44.84^ab^	44.92^ab^	4.15	0.015
Drip loss (%)	2.60	2.52	2.39	2.45	2.39	2.47	0.05	0.059
Cholesterol (mg/dl)	55.08^a^	55.01^a^	52.10^ab^	49.08^b^	53.32^ab^	52.76^ab^	3.16	0.046
Fat (%)	3.62^a^	3.55^a^	3.25^ab^	2.78^b^	3.26^ab^	3.30^ab^	0.45	0.036
ERV^$^ (mL)	13.05^b^	13.24^b^	15.85^a^	16.08^a^	14.37^ab^	14.44^ab^	1.67	0.002

T1 (no MOS/FOS/BMD), T2 (0.002% BMD), T3 (T1 + 0.1% MOS), T4 (T1 + 0.2% MOS), T5 (T1 + 0.1% FOS), T6 (T1 + 0.2% FOS).

SEM = Standard error of mean; NS = Non-significant; n = 15.

Mean values bearing the same superscript in a row did not differ significantly (*p* < 0.05).

^#^WHC = Water holding capacity, ^$^ERV = Extract release volume.

### Lipid oxidation parameters

The lipid peroxidation parameters are given in Table 4. The TBARS and free fatty acid (FFA) values revealed significant (*p* < 0.05) differences among the dietary treatments. The TBARS and FFA values of broiler chicken meat were lower in birds fed 0.2% MOS (T_4_ group) which did not differ significantly from other MOS, FOS, and BMD supplemented birds. The higher values were observed in control diet fed birds which were statistically to 0.1% MOS, BMD, and FOS supplemented birds. The peroxide values of chicken meat did not show any significant dietary effect.

**Table 4:** Effects of dietary inclusion of prebiotics on lipid oxidation parameter of fresh meat in broiler chickens.

Group parameters	T1	T2	T3	T4	T5	T6	SEM	*p* Value
TBARS* value (mg MDA**/kg)								
Breast	0.21^a^	0.19^ab^	0.18^ab^	0.16^b^	0.18^ab^	0.19^ab^	0.02	0.005
Thigh	0.16^a^	0.14^ab^	0.13^ab^	0.11^b^	0.14 ^ab^	0.13^ab^	0.03	0.031
*Free fatty acid (%)*								
Breast	0.006^a^	0.005^ab^	0.004^ab^	0.003^b^	0.005^ab^	0.004^ab^	0.002	0.017
Thigh	0.008^a^	0.006^ab^	0.006^ab^	0.004^b^	0.007^a^	0.006^ab^	0.03	0.024
*Peroxide value (meq/kg)*								
Breast	1.42	1.42	1.31	1.32	1.32	1.30	0.39	0.082
Thigh	1.29	1.30	1.28	1.26	1.27	1.25	0.57	0.071

T1 (no MOS/FOS/BMD), T2 (0.002% BMD), T3 (T1 + 0.1% MOS), T4 (T1 + 0.2% MOS), T5 (T1 + 0.1% FOS), T6 (T1 + 0.2% FOS).

Mean values bearing the same superscript in a row did not differ significantly (*p* < 0.05).

*TBRS = 2-Thiobarbituric acid reacting substances; MDA** = Malondialdehyd.

SEM = Standard error of mean; NS = Non-significant; n = 15.

### Antioxidant parameters

The results of antioxidant parameters affected by prebiotic supplementation in broiler chicken are given in Table 5. No significant differences were observed in ABTS values of breast and thigh meat among the dietary treatments. However, DPPH values of chicken breast and thigh meat were significantly (*p* < 0.05) higher in birds fed 0.2% MOS (T_4_ group) compared to control and BMD supplemented birds. But other MOS and FOS supplemented birds resulted in DPPH values similar to that of T_4_ group birds.

**Table 5:** Effects of dietary supplementation of prebiotics on anti-oxidant parameters of fresh meat in broiler chickens.

Group parameters	T1	T2	T3	T4	T5	T6	SEM	*p* Value
*ABTS + *(% inhibition)*								
Breast	87.24	86.38	87.95	87.38	87.19	86. 67	6.26	0.061
Thigh	79.77	69.05	89.05	79.81	69.57	69.05	5.43	0.245
**DPPH (% inhibition)								
Breast	20.55^b^	20.43^b^	22.54^ab^	24.33^a^	22.18^ab^	22.32^ab^	3.89	0.011
Thigh	13.96^b^	13.24^b^	14.78^ab^	16.69^a^	14.50^ab^	14.20^ab^	2.11	0.028

T1 (no MOS/FOS/BMD), T2 (0.002% BMD), T3 (T1 + 0.1% MOS), T4 (T1 + 0.2% MOS), T5 (T1 + 0.1% FOS), T6 (T1 + 0.2% FOS).

Mean values bearing the same superscript in a row did not differ significantly (*p* < 0.05).

ABTS +  = 2,2azino-bis -3-ethyl benzothiazoline-6-sulfonic acid; DPPH = 2, 2-diphenyl-1-picrylhydrazyl.

SEM = Standard error of mean; NS = Non-significant; n = 15.

### Microbial load

The results of microbial load of chicken meat as influenced by prebiotic supplementation are given in Table 6. In case of fresh meat (0 d), the levels of standard plate count (SPC), coliform, and staphylococci were significantly (*p* < 0.05) reduced in meat of birds supplemented with 0.2% (T_4_), 0.1% MOS (T_3_) and 0.2% FOS compared to birds fed control diet (T_1_) and antibiotics (T_2_). Whereas, at 14 d of storage, SPC were significantly decreased in both the MOS supplemented group, coliform counts were reduced (*p* < 0.05) in both the MOS and 0.1% FOS supplemented group but in case of staphylococci, significantly reduced in both the MOS and FOS dietary supplemented group compared to birds fed control and antibiotic diet. The MOS and FOS supplemented birds did not differ significantly from each other.

**Table 6:** Effects of dietary inclusion of prebiotics on microbial load of fresh and refrigerated (14 d) meat in broiler chickens.

Group parameters	T1	T2	T3	T4	T5	T6	SEM	*p* Value
*Microbial load (0d) (log*_*10*_*cfu/g)*								
Standard plate count	2.77^a^	2.50^a^	2.01^b^	2.32^b^	2.36^ab^	2.01^a^	0.03	0.021
*Coliform*	1.85^a^	1.25^ab^	1.05^b^	1.11^b^	1.10^b^	1.10^b^	0.04	0.002
*Staphylococcus aureus*	2.02^a^	1.32^ab^	1.19^b^	1.24^b^	1.29^ab^	1.24^b^	0.09	0.001
*Microbial load (14d) (log*_*10*_*cfu/g)*								
Standard plate count	3.61^a^	3.28^ab^	3.02^b^	3.05^b^	3.15^ab^	3.32^ab^	0.06	0.011
*Coliform*	2.36^a^	2.18^ab^	1.99^b^	2.06^b^	2.10^b^	2.16^ab^	0.03	0.032
*Staphylococcus aureus*	2.11^a^	1.64^ab^	1.33^b^	1.34^b^	1.38^b^	1.40^b^	0.05	0.006

T1 (no MOS/FOS/BMD), T2 (0.002% BMD), T3 (T1 + 0.1% MOS), T4 (T1 + 0.2% MOS), T5 (T1 + 0.1% FOS), T6 (T1 + 0.2% FOS).

SEM = Standard error of mean; n = 15.

Mean values bearing the same superscript in a row did not differ significantly (*p* < 0.05).

## Discussion

### Carcass traits

Similar to the results of present study, Toghyani et al.^14^ reported that carcass and cut-up parts yields were significantly higher in chicken fed prebiotic containing diet. However, in contrast to the present study Rehman et al.^15^ reported no significant differences in breast, thigh, and carcass yields after dietary inclusion of prebiotics. Whereas, Ricke^12^ observed no significant effect of prebiotics on the cut-up parts of chicken carcass. Therefore, based on the results of present study it can be assumed that the application of prebiotics has a positive effect on muscle weight. The principle effects of prebiotics have been reported by Cummings and Macfarlane^16^ and include improvement of calcium and magnesium absorption, production of short-chain fatty acids, and selective increases in the population of lactate producing bacteria like *Lactobacillus* and *Bifidobacterium*. It has been shown that increased lactate concentration often decreases intestine pH and is a potent anti-microbial substance to several pathogenic species such as *E.coli* ^17^. Thus, prebiotic helps to balance the intestinal microflora of poultry, consequently an improved utilization of diet nutrients i.e., protein and energy and higher feed intake leading to better cut up parts weight^14^.

### Physico-chemical indices

The results of the present study are in line with the findings of Pilarski et al.^18^, who reported that prebiotics caused a decrease in meat cholesterol concentration in comparison to the control and antibiotic treated group. In contrast, Salma et al.^19^ reported that no significant difference was observed in cholesterol concentration after dietary inclusion of prebiotics. The results of the present study were in accordance with the findings of Khaksefidi and Khaksefidi^20^, who observed that fat % of breast meat, was significantly lower in prebiotic supplemented chicken.

Fat deposition in the abdominal area of broilers is considered as waste in the poultry production; subsequently it represents a loss in the market and consumer acceptability, and increases expenditure during the treatment of effluent produced when processing broilers. The obtained results of this study indicate that prebiotic supplementation of broilers diet has the potential to lessen this type of waste by reduction of the fat content in the abdominal area of birds^14^.

In the present study, the pH values were within the normal range and independent of dietary prebiotic supplementation. Similar to the results of the present study Tavaniello et al.^13^ did not find any significant effect of dietary prebiotic supplementation on the pH values of chicken meat. However, Mir et al.^21^ confirms that the meat quality is influenced by pH changes which occur during rigor mortis. Generally meat with high pH has high WHC, although the present study does not support this correlation. The results of present study are in line with Habibi-Najafi et al.^22^, who reported that dietary supplementation of prebiotic increased the WHC of meat. On the other hand, Harriet et al.^23^ reported that dietary inclusion of prebiotic has no significant effect on WHC of meat during storage condition. It is remarkable to note that water loss reduces meat nutritional value because some nutrients may be lost in exudate resulting in meat becoming less tender and bad in flavour. Regarding ERV values in broiler chicken after the dietary inclusion of FOS and MOS, no such reports are available for comparing the results of this study.

### Lipid oxidation parameters

The results of present study showed that prebiotic could inhibit both thigh and breast muscle lipid oxidation (MDA production) in broiler chicken, therefore protecting the peroxidation of labile PUFA enriched meat. The reduced shelf-life of meat occurs due to progressive oxidation and enzymatic hydrolysis of unsaturated fatty acid^24^ FFA value is the measure of hydrolytic rancidity due to lipolytic enzyme activity of microbial and muscle origin resulting in accumulation of FFA which might impart undesirable flavour in foods^25^. The peroxide value test involves the measurement of peroxide and hydro peroxide formed during initial stage of lipid oxidation^26^. However, in contrast to the results of present study Konca et al.^27^ reported that after the dietary inclusion of prebiotics, TBARS values were significantly increased. Furthermore, Ali^28^ reported that dietary inclusion of prebiotics has no pivotal role in changing the TBARS activities in fresh as well as stored meat. No clear mechanisms have been reported responsible for the reduction of lipid synthesis by prebiotics. It might in part be due to increasing beneficial bacteria such as *Lactobacillus* that decrease the activity of acetyl-CoA carboxylase, which is the rate-limiting enzyme in fatty acids synthesis^14^.

### Antioxidant parameters

The natural dietary antioxidant compounds of plant origin react with lipids and hydroxyl radicals and result into stable product. Simitzis et al.^29^ reported that following absorption prebiotics have shown significant antioxidant activity in poultry meat after entering the systemic circulation. The lipid and cholesterol oxidation of broiler chicken meat was significantly reduced by dietary prebiotic supplementation in broiler chicken^30^. Inclusion of prebiotics in turkey diet increased the oxidation stability and retention of alpha tocopherol in the long term stored frozen turkey meat^31^. It is still unclear whether the dietary antioxidants consumed can be incorporated into fatty tissues in the same form as when the fat is stabilized in-vitro^32^. However in the present study, free radical inhibition percentage of thigh and breast meat of chicken fed 0.2% MOS was significantly greater than that of chicken fed control and antibiotic supplemented diet. These results indicate that antioxidant compounds from prebiotic prevented thigh and breast meat from oxidation.

### Microbial load

According to the hypothesis proposed by Kim et al.^33^ the reduction in microbial load was due to production of different antimicrobial components by prebiotic which result in exclusion of common entero-pathogens and food spoilage organisms of broiler chicken. Though, the exact mechanism by which prebiotics might exert anti-microbial effects in broiler chicken meat remains unclear. Some of the proposed modes of actions are; maintaining a healthy balance of gut microflora, competitive exclusion and inhibition of microbial growth by lactic acid producing bacteria favoured by dietary prebiotics, enhancing gut immunity and integrity, improving digestive enzyme activities, digestion and neutralizing enterotoxins, etc.^34^. It is general hypothesis that prebiotics have been shown to alter gastrointestinal microflora, modify the immune system, reduce pathogen annexation including pathogens such as *Salmonella Entritidis* and *E.coli*^16^. Prebiotics supplementation of broilers diet also result in an increase of the pH of the gastro intestinal tract (GIT) and beneficial bacteria population such as *lactobacillus* and *bifidobacterium*, due to increasing production of volatile fatty acids^35^.

## Conclusions

The results reported in this work indicate that 0.2% mannan oligosaccharides (MOS) could be used as natural growth promoter (NGP) to replace the antibiotic growth promoter (AGP) in improving the physico-chemical, oxidative stability, and microbiological quality of broiler chicken meat. Subsequent the appropriate guidelines and protocols will ensure eventually limited the use of feed antibiotic for poultry production and the induction of NGP in animal derived food products i.e., meat which will reduce the risk to the public. This NGP could be popularized among the farmers as a feed additive in poultry diets for production of safe, clean, and green poultry meat for human consumption.

## Material and methods

### Animal ethics compliance

This study was approved and carried out according to the guidelines of Institutional Animal Ethics Committee (IEAC) of ICAR-Central Avian Research Institute, Izatnagar. The study was carried out in compliance with the Animal Research: Reporting of in Vivo Experiments (ARRIVE) guidelines.

### Birds, housing and feeding

A total of 240 day-old commercial broiler chickens of uniform body weight were used in this study. The birds were divided randomly into 6 treatment groups with 5 replicate each and having 8 birds in each replicate with equal number of males and females, distinguished by vent sexing method. The birds were reared under uniform standard managemental conditions in electrically heated battery brooders (12 ft^2^ for 8 birds i.e., 1.5 ft^2^ per bird) and birds were vaccinated following the routine vaccination schedule of our experimental farm. The birds were provided 24 h light for first three days followed by a decrease of 1 h per day till it reached 18 h light period which was continued till the end of trial. The initial cage temperature was 35 °C which was reduced by 1 °C every week to provide thermo-comfort environment to the birds.

### Experimental diets

Bacitracin methylene di-salicylate (BMD), with a certified 44% bacitracin activity, was purchased from ALPHARMA Animal Health Division New Jersey, USA. Mannan oligosaccharides (MOS) and Fructo-oligosaccharides (FOS) were purchased from M/s Kothari Fermentation and Biochem Ltd., India and National Dairy Research Institute, Karnal India respectively. Six iso-caloric and iso-nitrogenous corn-soya primarily based (Table 7) dietary treatments were formulated viz. T_1_ (control diet), T_2_ (T_1_ + bacitracin methylene di-salicylate @ 0.002%), T_3_ (T_1_ + 0.1% MOS), T_4_ (T_1_ + 0.2% MOS), T_5_ (T_1_ + 0.1% FOS), and T_6_ (T_1_ + 0.2% FOS). The birds were provided ad libitum respective feed and fresh water throughout the feeding trial of 42 days.

**Table 7:** Composition of basal diet where dietary supplementation of prebiotics were added.

Ingredients (g/kg)	Starter (0–21d)	Finisher (21–424d)
Maize	549.55	615.85
Soybean	382.00	320.00
RSM	30.00	30.00
Oil	6.00	4.00
Limestone	9.00	9.00
DCP	16.00	14.00
Salt	3.00	3.00
DL-Methionine	1.30	1.00
*TM Premix-1	1.00	1.00
**Vit Premix-2	1.50	1.50
***B. Complex	0.15	0.15
Ch. Chloride	0. 50	0.50
*Calculated value*		
ME (Kcal/kg)	2952.31	3000.35
Crude protein (%)	21.50	19.51
Total phosphorus (%)	0.43	0.38
Total calcium (%)	0.98	0.91
Lysine (%)	1.20	1.06
Methionine	0.50	0.45
Threonine	0.95	0.86

*Premix 1: Each g of mineral mixture contained: 200 mg of FeSO_4_·7H_2_O, 20 mg of CuSO_4_·5H_2_O, 200 mg of MnSO4.H2O, 150 mg of ZnSO_4_.7H_2_O, 1 mg of KI.

**Premix 2: Each g of vitamin A, B_2_, D_3_, K (Spectromix, Ranboxy) provided: vitamin A (retinol)-540 mg, vitamin B2 (riboflavin)-50 mg, vitamin D_3_ (cholecalciferol)-400 mg, vitamin K (menadione)-10 mg.

***B. Complex*:* Each g of B-complex provided: vitamin B1 (thiamine)-2 mg, folic acid-10 mg, pyridoxine HCl-4 mg, cyanocobalamin-10 µg, nicotinamide-12 mg.

### Carcass characteristics

Toward the finish of 42 days trial period, 15 birds from every treatment (three birds for each replicate) were electrically stunned (200 V applied for 3 s) and slaughtered by exsanguination after 12 h of fasting with ad libitum drinking water. The carcass characteristics (dressing and eviscerated yield), cut up parts (thigh, breast, back, wings and drumstick) and relative weight of organs (heart, liver, and gizard**)** were determined.

### Collection of sample

The breast and thigh meat samples were collected separately from every slaughtered bird for the study of physico-chemical, oxidative stability, and microbial characteristics.

### Physico-chemical indices

Fat content (percentage, dry basis) of meat was determined by refluxing 2 g dried meat sample in 150 mL petroleum ether in Soxhlet extraction equipment for 6 h at 60°C^36^. For cholesterol estimation about 1 g meat sample was extracted in 15 mL chloroform methanol mixture (2:1) and the concentration of cholesterol within the extract was determined by spectrophotometer at wavelength of 560 nm^26^. The pH of meat sample was measured with the assistance of digital pH scale meter by mixing 5 g meat sample with 25 mL distilled water for 2 min^21^. For the estimation of purge loss/drip loss, the frozen meat samples were weighed and recorded as the initial weight (W1). The weighed samples were placed into polyethylene bags, labelled, and keep hanging at 4 °C for 24 h. The meat samples were weighed once more and final weight (W2) was recorded. Drip loss was calculated as shown in the equation below:

$${\text{Drip}}\;{\text{loss}}\left( \% \right) = \left[ {\left( {{\text{W}}1 - {\text{W}}2} \right)/{\text{W}}1} \right] \times 100.$$

To determine the extract release volume (ERV) of meat samples, 15 g samples were blended with 60 mL phosphate buffer solution (0.05 M; pH 5.8) for two minutes and the homogenate was filtered through Whatman filter paper No. 1 for a fixed time period of 15 min to the filtrate measured as ERV^37^. Water holding capacity (WHC) of meat samples was determined by mixing 10 g minced meat sample in 15 mL of 0.6 M NaCl for 2 min followed by refrigerated (4 °C) holding for 15 min. The slurry is then shaken, centrifuged at 5000 RPM for 15 min, the supernatant fluid was decanted and measured^38^.

$${\text{WHC}}\left( \% \right) = \left[ {\left( {{\text{vol}}.\;{\text{of}}\;{\text{NaCl}}\;{\text{added}}{-}{\text{vol}}.\;{\text{of}}\;{\text{supernatant}}} \right)/{\text{weight}}\;{\text{of}}\;{\text{sample}}} \right] \times 100.$$

### Lipid peroxidation parameters

The lipid peroxidation was determined by estimating the thio-barbituric acid reactive substance (TBARS) in the selected meat sample. About 5 g meat sample was extracted in 12.5 mL 20% TCA (made in 2 M orthophosphoric acid) solution for 2 min and the slurry was mixed with 12.5 mL cold distilled water followed by filtration through Whatman paper No. 1. Then 3 mL of filtrate was mixed with 3 mL of TBA reagent (0.005 M), mixture was kept in dark cabinet for 16 h and absorbance (O.D) was measured by a spectrophotometer (UV/VIS, Varian, make up of spectrophotometer) at fixed wavelength of 532 nm against the blank made by mixing of 3 mL of 10% TCA and 3 mL of TBA reagent^39^. TBARS value was calculated as mg malonaldehyde (MDA) per Kg of sample by multiplying O.D value with K-factor of 5.2.

The free fatty acid value and peroxide value was determined in the selected meat sample. About 5 g meat sample was blended with 30 mL chloroform for 2 min in presence of anhydrous sodium sulphate powder followed by filtration into conical flask through No. 1 Whatman paper^40^. For free fatty acid value about 2–3 drops of 0.2% phenolphthalein indicator was added to the chloroform extract followed by titration with 0.1 N alcoholic potassium hydroxide to get the pink colour end point. For peroxide value 30 mL of glacial acetic was added to 25 mL of chloroform extract, then 2 mL potassium iodide solution was added, and the mixture was allowed to stand for 2 min with occasional shaking. Then, 100 mL distilled water and 2 mL fresh 1% starch solution were added to the mixture following titration with 0.1 N sodium thiosulphate till the end point was reached (non-aqueous layer turned colourless). The calculations were made as follows:

$${\text{Free}}\;{\text{fatty}}\;{\text{acid}}\left( \% \right) = \left[ {\left( {0.1 \times {\text{vol}}.\;{\text{of}}\;{\text{KOH}}\;{\text{consumed}} \times 0.282} \right)/{\text{sample}}\;{\text{weight}}} \right] \times 100.$$

$${\text{Peroxide}}\;{\text{value}}\left( {\text{meq/kg}} \right) = \left[ {\left( {0.1 \times {\text{vol}}.\;{\text{of}}\;{\text{sodium}}\;{\text{thiosulphate}}\;{\text{consumed}}} \right)/{\text{sample}}\;{\text{weight}}} \right] \times 1000.$$

### Antioxidant parameters

About 5 g meat sample was triturated in 20 mL ethanol for 2 min followed by filteration through Whatman paper No. 42. For ABTS^+^ (2, 2-azinobis-3-ethylbenzothiazoline-6- sulfonic acid) assay 2 mL of ABTS working solution (7 mM) was added to 1 mL filtrate and absorbency was measured by spectrophotometer (UV/VIS, Varian, make up of spectrophotometer) at fixed wavelength of 734 nm after 20 min (At_20_)^41^. For DDPH (1, 1-diphenyl-2- picrylhydrazyl) assay 1 mL filtrate was mixed with 1 mL 0.1 M Tris–HCl buffer (pH 7.4) and 1 mL DPPH reagent (250 µM). The absorbency was measured immediately (At_0_) and after 20 min (At_20_) by spectrophotometer (UV/VIS, Varian) at fixed wavelength of 517 nm^42^. The calculations were made as follows:

$${\text{ABTS}}\;{\text{activity}}\left( {\% \;{\text{inhibition}}} \right) = \left[ {\left( {0.7{-}{\text{At}}_{20} } \right)/0.7} \right] \times 100$$

$${\text{DPPH}}\;{\text{activity}}\left( {\% \;{\text{inhibition}}} \right) = 100 - \left( {{\text{At}}_{20} /{\text{At}}_{0} } \right) \times 100$$

### Microbial load

The microbial load of the meat samples were estimated in terms of specific plate count (SPC), coliform count, and staphylococcus count. About 1 g sample was homogenized with 10 mL of 0.1% peptone water (Hi-media, make up of all agars used in this study) with the aid of sterile pestle and mortar under aseptic condition to give a 10:1 initial dilution. The homogenate was used for the preparation of tenfold serial dilution up to 10^6^:1 with 0.1% peptone water in sterile test tubes. One mL aliquot of each dilution was placed in identified sterile petri dishes aseptically. About 12–15 mL of sterile molten and cooled (45 °C) specified agar (Himedia) was poured on each petri dish and mixed gently. After setting, the plates were incubated at 37 °C for 48 h and colonies were counted using a Quebec colony counter. The counts were multiplied by the respective dilution and calculated per gram of sample as log_10_ cfu.

### Statistical analysis

The experimental unit for the data analysis was the sampled bird. Prior to the analysis, all the data were tested for normality and homogeneity of variances with the Shapiro–Wilk test and Levene's test, respectively. The data were analysed by one way ANOVA by using the General Linear Model procedure (IBM SPSS software-20). However, data of the measurements repeated after 14 days were subjected to mixed model procedure for repeated measure analysis. The Tukey post-hoc analysis was done to test the significant mean differences between the groups with significance level defined at *p* < 0.05.

### Ethical approval

All applicable institutional guidelines for the care and use of animals were followed. The experimental procedures carried out in this study were approved by the Institutional Animal Ethics Committee (IEAC) following the guidelines of ‘Committee for the Purpose of Control and Supervision of Experiments on Animals (CPCSEA) 2012’established under the “Prevention of Cruelty of Animals Act 1960” of Indian Penal Code (18 September 2017/Project No. 11). The study was carried out in compliance with the Animal Research: Reporting of in Vivo Experiments (ARRIVE) guidelines.

## Data Availability

The datasets analysed during the current study are available from the corresponding author on reasonable request.
